# Supplementary material for: Altered Neocortical Gene Expression, Brain Overgrowth and Functional Over-Connectivity in Chd8 Haploinsufficient Mice
Source: Cereb Cortex. 2018 Apr 13;28(6):2192–206. doi: 10.1093/cercor/bhy058 (PMC6018918; doi:10.1093/cercor/bhy058)
Supplement: Supplementary Data [file bhy058suppl_1.zip › Suetterlin_supplementary_table_legends.docx]

**Supplementary Tables:**

Supplementary Table 1: Absolute and relative volumetric differences in specific brain regions between *Chd8^+/-^* and *Chd8^+/+^* mice as determined by MRI.

Supplementary Table 2: Differentially expressed genes in E12.5 *Chd8^+/-^* neocortices compared to wildtype controls.

Supplementary Table 3: Differentially expressed genes in P5 *Chd8^+/-^* neocortices compared to wildtype controls.

Supplementary Table 4: SFARI ASD genes overlapping with P5 differentially expressed genes.

Supplementary Table 5: Up-regulated Gene Ontology: Biological Processes

Supplementary Table 6: Down-regulated Gene Ontology: Biological Processes

Supplementary Table 7: Up-regulated Gene Ontology: Molecular Function

Supplementary Table 8: Down-regulated Gene Ontology: Molecular Function

Supplementary Table 9 Up-regulated Gene Ontology: Pathways

Supplementary Table 10: Down-regulated Gene Ontology: Pathways

Supplementary Table 11: Gene expression enrichment analysis and Gene Ontology analysis in hippocampal CA2 and auditory areas.
