# Supplementary material for: Altered Neocortical Gene Expression, Brain Overgrowth and Functional Over-Connectivity in Chd8 Haploinsufficient Mice
Source: Cereb Cortex. 2018 Apr 13;28(6):2192–206. doi: 10.1093/cercor/bhy058 (PMC6018918; doi:10.1093/cercor/bhy058)
Supplement: Supplementary Data [file bhy058suppl_1.zip › SupplementaryFigurelegends_2_final.docx]

**Supplementary Figure 1. Construction and validation of the *Chd8* conditional and null alleles.**

A) Diagrammatic representations of the wildtype (wt) *Chd8* locus (*Chd8^wt^*), targeting construct, targeted (neo), conditional (flox) and null alleles. Approximate genomic distances are indicated in kilobases (kb), exons are denoted by boxes labelled Ex1 to 13, and Southern blot probes (P1,P2) and PCR primers (#1- #4) are indicated. The 5’ long homology arm is shown in red and the 3’ short homology arm in green. The neomycin resistance cassette (neo) is shown as a blue box, the floxed exon 3 by a black box, loxP sites by red triangles and frt sites by tan semi-ovals. BclI restriction enzyme sites are labelled.

B) Southern blot of genomic DNA digested with BclI from embryonic stem (ES) cell clones (123, 124 etc.) and wildtype C57BL/6J (B6) cells, hybridised with P1 are shown, with molecular weight markers in the left hand lane. The wildtype allele (WT) gives a 24kb band, whilst the targeted allele gives a band of approximately 10.4kb. Southern blot of genomic DNA digested with BclI from embryonic stem (ES) cell clones as indicated and wildtype B6 cells, hybridised with P2 are shown, with molecular weight markers in the left hand lane. The wildtype allele (WT) gives a 24kb band, whilst the targeted allele gives a band of approximately 15.3kb.

C) PCR genotyping of genomic DNA extracted from mouse pups from a cross between a heterozygous general deleter *βactin-Cre* transgenic mouse and a *Chd8^flox/flox^* mouse. Results from PCR reactions to detect the Cre transgene, distinguish the *Chd8^flox^* and wildtype alleles from each other, and amplify the null allele are shown. Note the loss of the flox allele, with the gain of the null allele in the Cre^+^ pup (lane 4).

D) Quantitative RT-PCR for *Chd8* on mRNA extracted from *Chd8* heterozygous mouse neocortices at E12.5 and P5 and littermate controls using primers spanning the exon3-4 boundary. *Chd8* expression levels in heterozygous mice are significantly reduced to 36% of wildtype controls at E12.5 (**p=0.0059, t=5.346, df=4, student’s t-test, n=3 per genotype) and 48% at P5 (*p=0.0105, t=4.538, df=4, student’s t-test, n=3 per genotype).

E) Western blot on lysates from *Chd8* heterozygous E12.5 neocortices and littermate controls. Upper panel: The band for full-length CHD8 (arrow, ~290kDa) was quantified in F. Note the absence of detectable levels of truncated protein products for CHD8. Lower panel: Western blot for the loading control GAPDH.

F) Quantification of CHD8 protein levels normalised to GAPDH as shown in E. CHD8 protein levels in heterozygous mice is 49% compared to wildtype littermates (p=0.156, student’s t-test, n=3 per genotype).

G) Quantitative RT-PCR for *Chd8* on mRNA extracted from *Chd8* heterozygous mouse neocortices at E12.5 and littermate controls using primers spanning the exon1-2 boundary (p=0.116, student’s t-test, n=3 per genotype). Samples are the same as in D.

**Supplementary Figure 2: Functional Enrichment Analysis of differentially expressed genes (DEGs) in *Chd8^+/-^* P5 neocortices.**

A) Results of gene set enrichment analysis using the DAVID knowledgebase on the P5 DEG set (FDR < 0.05). The five most significantly enriched KEGG pathways are shown for up-regulated DEGs (top left panel) and downregulated DEGs (top right panel), respectively. The five most significant Gene Ontology terms in the Molecular Function category are shown for up-regulated DEGs (bottom left panel) and downregulated DEGs (bottom right panel).

B) DEGs (FDR < 0.05) were subjected to enrichment analysis using the “ENCODE and ChEA Consensus TFs from ChIP-X” database on Enrichr, to identify putative upstream regulatory transcription factors. The four most overrepresented transcription factors are shown for up-regulated (left) and down-regulated (right) DEGs, respectively.

**Supplementary Figure 3: Linear regression analysis of regional brain volumes and activity levels in adult male mice.**

Activity levels in the open field test were plotted against hippocampal volume (A), cortical volume (B) and brain volume (C) in individual male mice (*Chd8^+/+^*, n=12; *Chd8^+/-^*, n=11). Linear regression analysis: R^2^=0.264, p=0.012 (hippocampus), R^2^=0.193, p=0.036 (cortex), R^2^=0.129, p=0.092 (brain).

**Supplementary Figure 4: Gene expression enrichment analysis in hippocampus (CA2) and auditory areas.**

(A) Intersection of genes with high relative expression in the CA2 region and auditory areas. High relative expression was defined as being in the top 20% of genes in a given area.

(B) Genes colour coded by their summed relative expression (L1 distance) in CA2 and auditory areas.

**Supplementary Figure 5: Anaesthesia sensitivity in *Chd8^+/-^* mice and wildtype littermate controls.**

(A) Continuous monitoring of arterial blood pressure in *Chd8^+/-^* mice and *Chd8^+/+^* littermate controls. Blood pressure does not differ significantly between groups (p=0.79, t=0.274, df=40; student’s t-test).

(B) Mean BOLD amplitude in motor cortex of *Chd8^+/-^* mice and *Chd8^+/+^* littermate controls. Mean BOLD amplitude does not differ significantly between groups (p=0.56, t=0.535, df=40; student’s t-test).
