# Supplementary figures and images for: Altered Neocortical Gene Expression, Brain Overgrowth and Functional Over-Connectivity in Chd8 Haploinsufficient Mice
Source: Cereb Cortex. 2018 Apr 13;28(6):2192–206. doi: 10.1093/cercor/bhy058 (PMC6018918; doi:10.1093/cercor/bhy058)

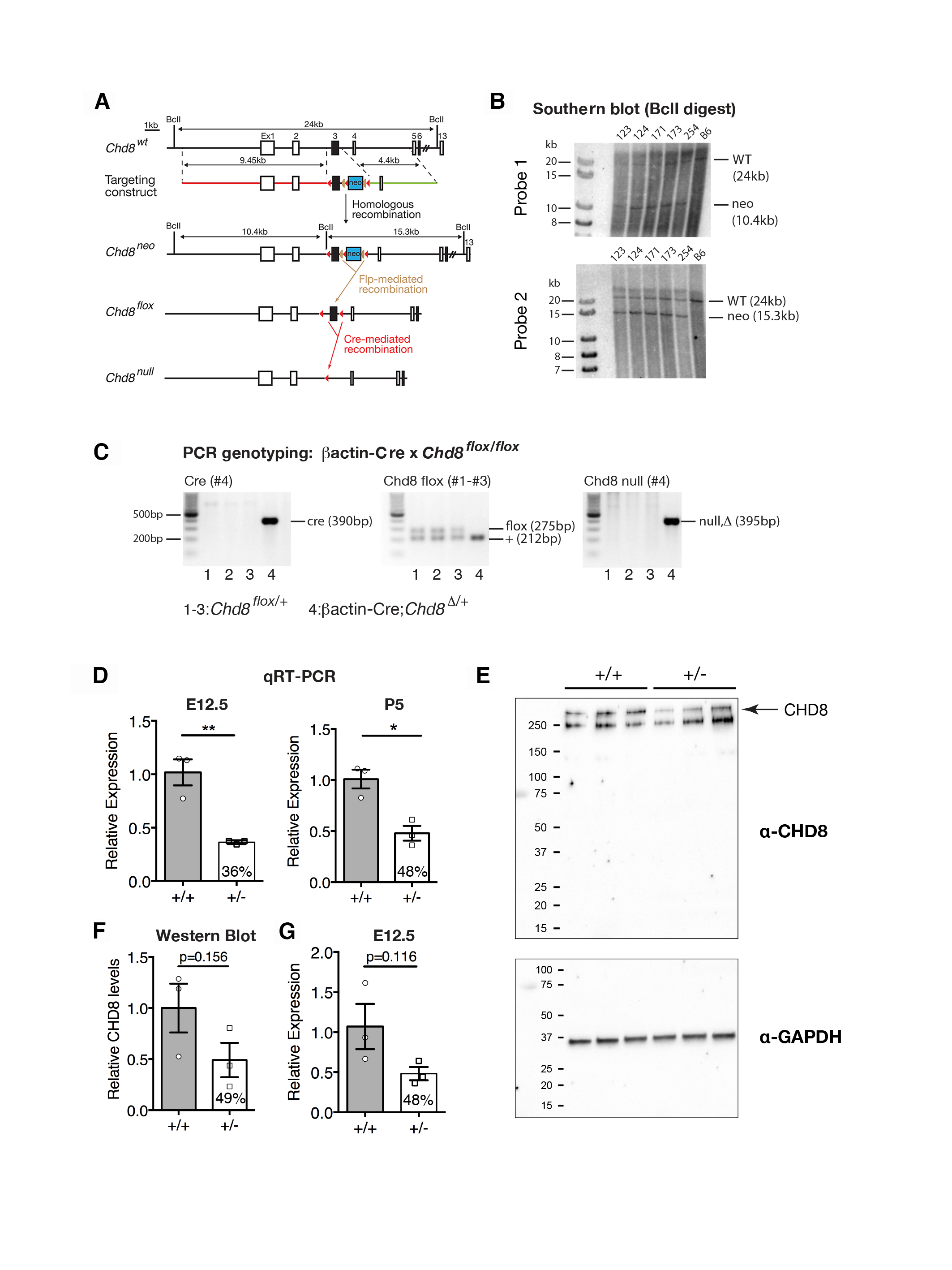

Supplement: Supplementary Data [file bhy058suppl_1.zip › FigureS1_corrected.png]

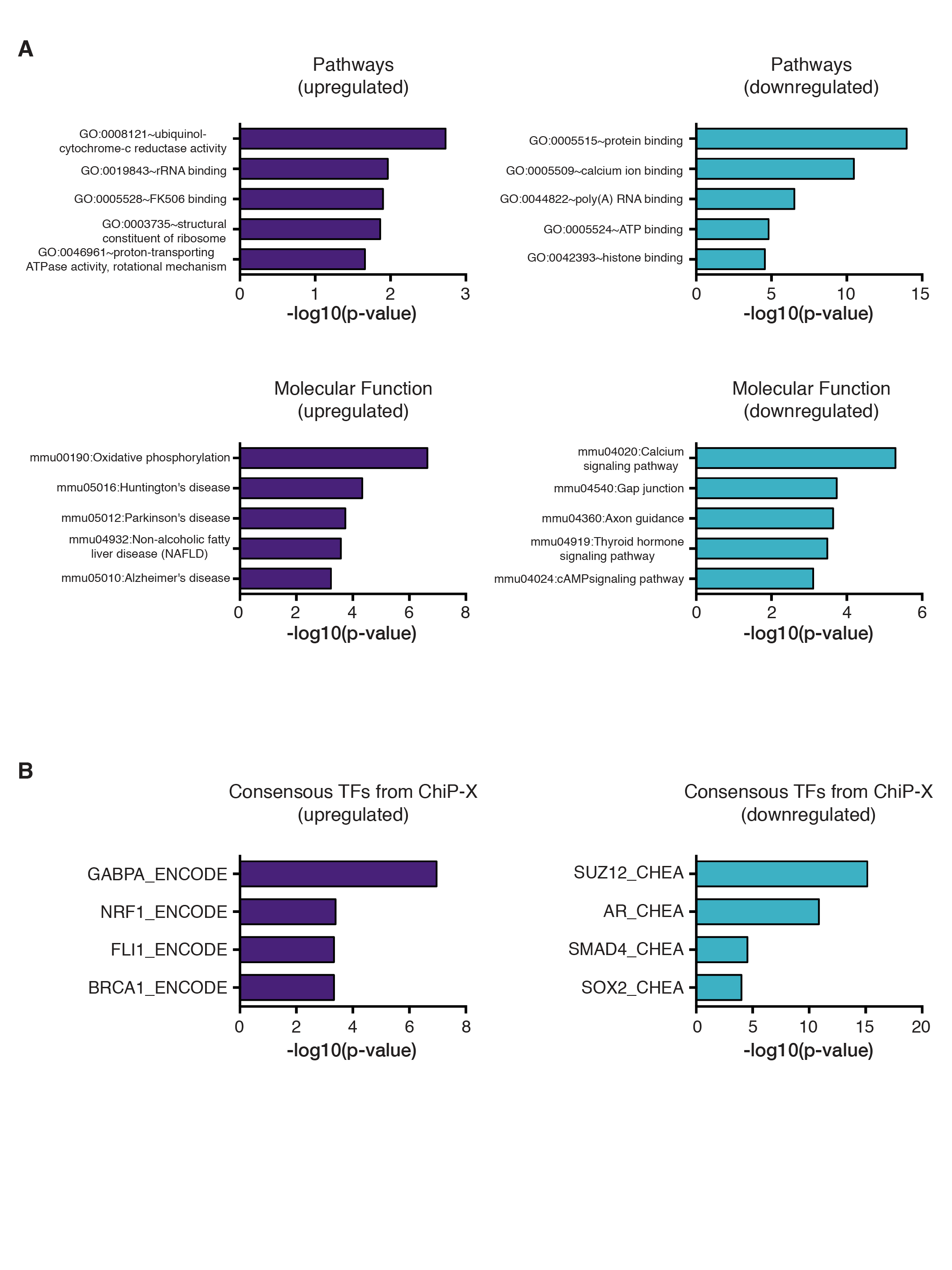

Supplement: Supplementary Data [file bhy058suppl_1.zip › FigureS2.png]

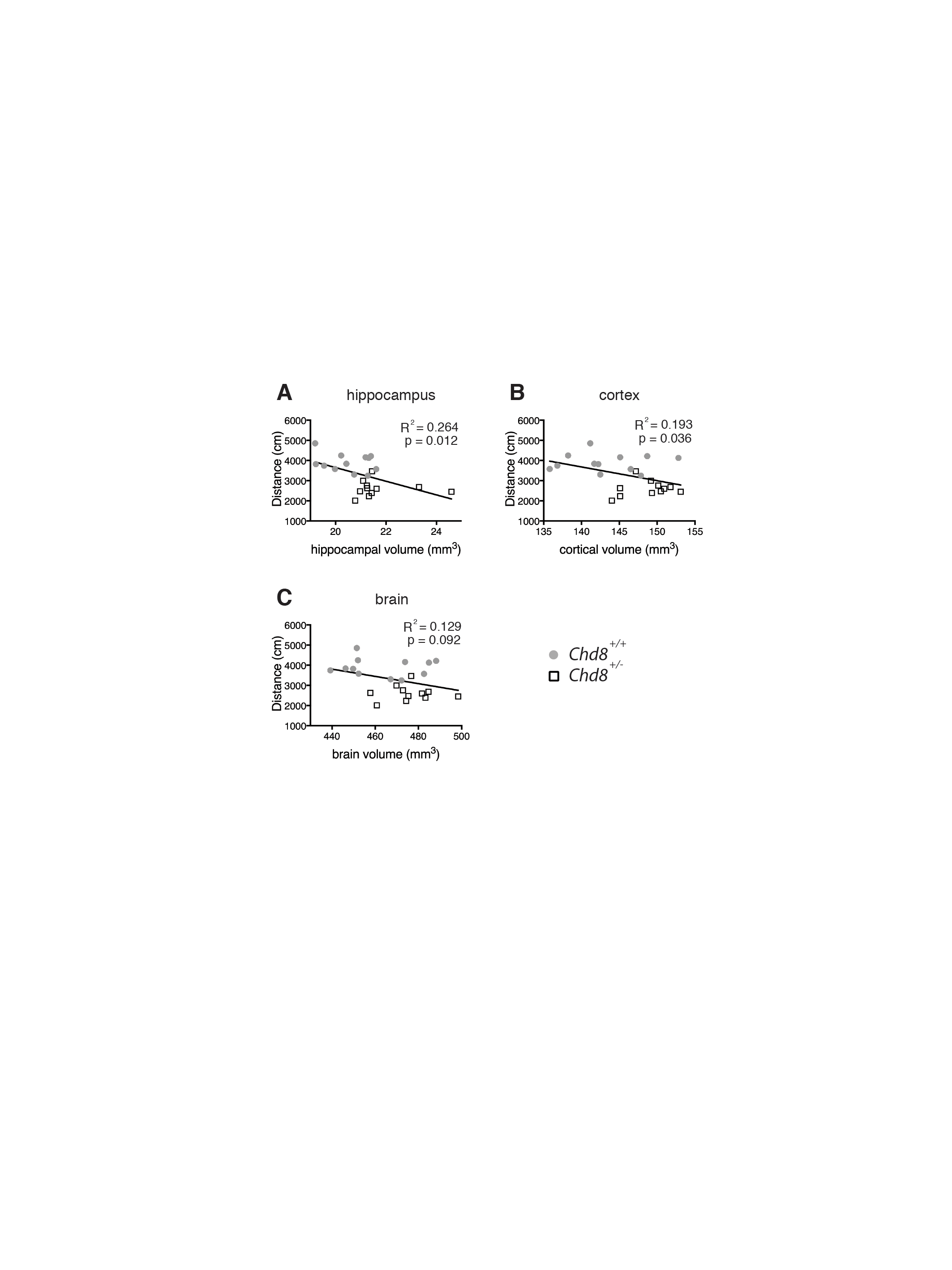

Supplement: Supplementary Data [file bhy058suppl_1.zip › FigureS3.png]

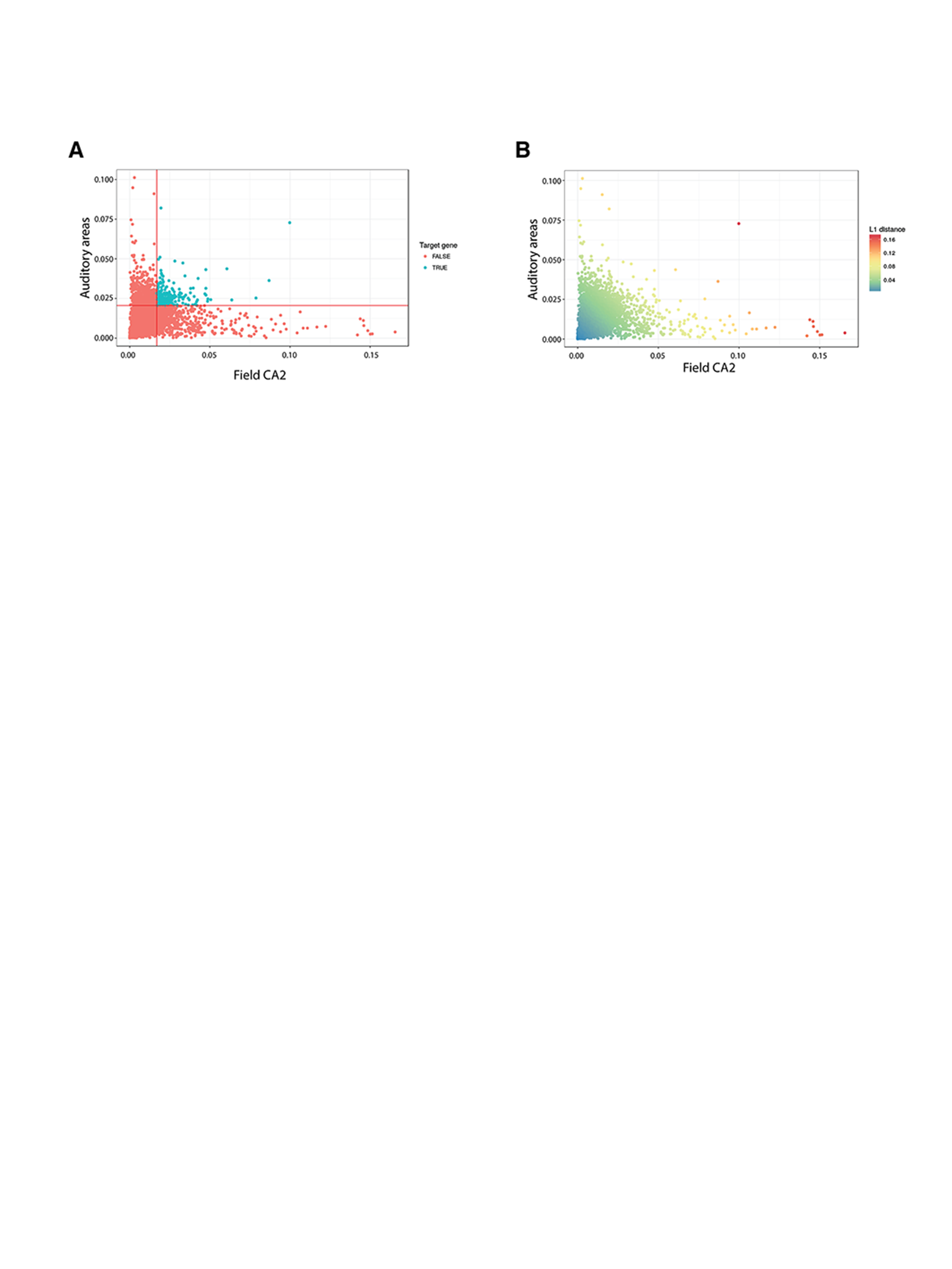

Supplement: Supplementary Data [file bhy058suppl_1.zip › FigureS4_corrected.png]

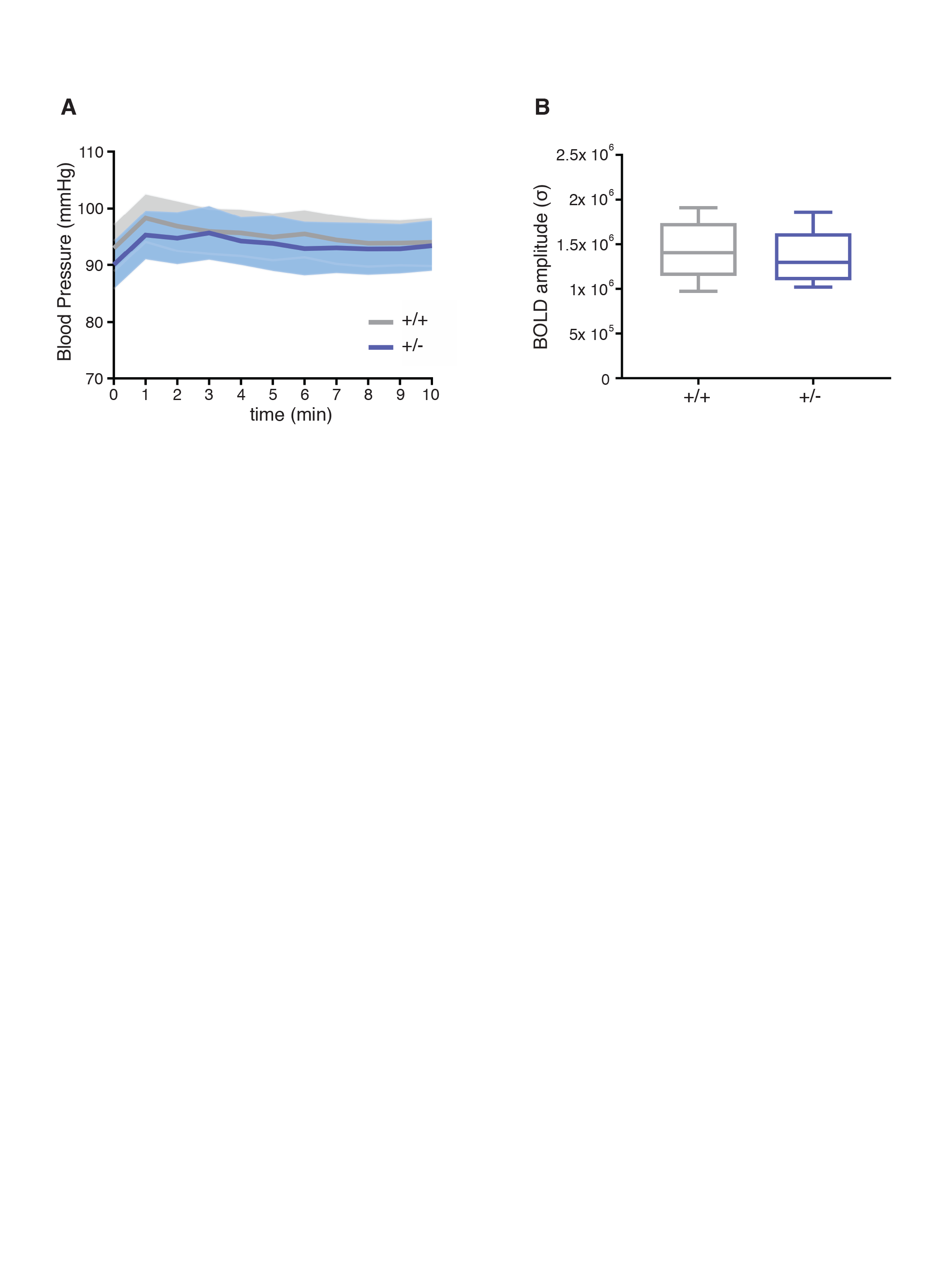

Supplement: Supplementary Data [file bhy058suppl_1.zip › FigureS5.png]
